# Melatonin Attenuates Dextran Sodium Sulfate Induced Colitis in Obese Mice

**DOI:** 10.3390/ph14080822

**Published:** 2021-08-21

**Authors:** Shijia Pan, Fan Hong, Letong Li, Yuan Guo, Xiaoxiao Qiao, Jia Zhang, Pengfei Xu, Yonggong Zhai

**Affiliations:** 1 Beijing Key Laboratory of Gene Resource and Molecular Development, College of Life Sciences, Beijing Normal University, Beijing 100875, China; 201931200003@mail.bnu.edu.cn (S.P.); hongfanky@126.com (F.H.); 201921200016@mail.bnu.edu.cn (L.L.); gy5326@126.com (Y.G.); 201921200023@mail.bnu.edu.cn (X.Q.); 202021200029@mail.bnu.edu.cn (J.Z.); 2 Key Laboratory for Cell Proliferation and Regulation Biology of State Education Ministry, College of Life Sciences, Beijing Normal University, Beijing 100875, China; 3 Center for Pharmacogenetics and Department of Pharmaceutical Sciences, University of Pittsburgh, Pittsburgh, PA 15261, USA

**Keywords:** melatonin, obesity, colitis, lipolysis, autophagy

## Abstract

Epidemiological studies have indicated that obesity is an independent risk factor for colitis and that a high-fat diet (HFD) increases the deterioration of colitis-related indicators in mice. Melatonin has multiple anti-inflammatory effects, including inhibiting tumor growth and regulating immune defense. However, the mechanism of its activity in ameliorating obesity-promoted colitis is still unclear. This study explored the possibility that melatonin has beneficial functions in HFD-induced dextran sodium sulfate (DSS)-induced colitis in mice. Here, we revealed that HFD-promoted obesity accelerated DSS-induced colitis, while melatonin intervention improved colitis. Melatonin significantly alleviated inflammation by increasing anti-inflammatory cytokine release and reducing the levels of proinflammatory cytokines in HFD- and DSS-treated mice. Furthermore, melatonin expressed antioxidant activities and reversed intestinal barrier integrity, resulting in improved colitis in DSS-treated obese mice. We also found that melatonin could reduce the ability of inflammatory cells to utilize fatty acids and decrease the growth-promoting effect of lipids by inhibiting autophagy. Taken together, our study indicates that the inhibitory effect of melatonin on autophagy weakens the lipid-mediated prosurvival advantage, which suggests that melatonin-targeted autophagy may provide an opportunity to prevent colitis in obese individuals.

## 1. Introduction

Accumulated evidence has indicated a consistent and compelling association between obesity and the risk of colitis development [1,2]. The etiology of colitis is generally associated with abnormal mucosal barrier function, immune system dysfunction, and environmental factors. Excessive intake of westernized diets could promote obesity but also increase the risk of worsening inflammation, aggravating colitis [3,4]. Excessive deposition of mesenteric white adipose tissue (Mes-WAT) in obese individuals causes hypertrophic adipocytes to release different pro-inflammatory adipokine and chemokine complement factors. They could affect the integrity and permeability of the intestinal mucosa and disrupt immune homeostasis in the intestine, which in turn leads to upregulation of adipose-derived inflammatory cytokines, bacterial translocation, and macrophage aggregation, making them susceptible to colitis [5,6]. Long-term colitis can easily worsen into colorectal cancer (CRC) [7,8], and the risk of CRC in patients with colitis is higher than that in the general population. A key measure is chemoprevention [9]; that is, a strategy aimed at inhibiting obesity-induced inflammation would benefit the prevention of inflammatory bowel disease (IBD) and CRC. Therefore, it is particularly important to find medicines with few side effects that solve the fundamental problem of anti-inflammation to reduce the risk of IBD and CRC.

Melatonin (N-acetyl-5-methoxytryptamine) is an endogenous hormone mainly secreted by the pineal gland. Its function is to regulate circadian rhythms, immunity, and antioxidants [10,11,12]. Cumulative evidence has discussed that melatonin has a wide range of protective effects on various diseases, such as arrhythmia [13], primary biliary cholangitis [14], diabesity [15], non-small cell lung cancer [16], anovulatory disorders [17], cutaneous diseases [18], neurodegenerative diseases [19,20], and autism [21]. Furthermore, the beneficial effects of melatonin have also been studied in *Sparus aurata* [22] and *Plasmodium falciparum* [23]. In these studies, melatonin acted as a protector by regulating pathophysiological mechanisms and signaling pathways. It would explain the question of why melatonin might be used in adjuvant therapy for some diseases. Interestingly, Melatonin is also produced by enterochromaffin cells (ECs) in the gastrointestinal tract (GIT), where its level is 400 times higher than that of the pineal gland [24]. The secretion is associated with food intake [25]. After the addition of exogenous melatonin, the most obvious melatonin accumulation was observed in the colon and rectum [26]. Subsequent studies have also verified that melatonin secretion is widespread in the GIT and it has a wide range of melatonin binding sites, suggesting that it is related to the complex regulation of gastrointestinal physiology. Interestingly, it has been suggested that a decrease in the release and distribution of endogenous melatonin might be related to the pathogenesis of colitis [27,28]. The loss in melatonin availability was associated with an increasing extent of intestinal damage [24]. Moreover, circadian rhythm disruption is acted as a risk factor for colitis. A disrupted circadian rhythm would worsen colitis and reduce the level of melatonin in DSS-induced colitis mice [29]. Epidemiological studies have also shown that sleep disturbances in IBD patients, is a type of circadian rhythm disruption and an environmental predictor factor for the worsening of IBD [30,31]. Some animal trials and clinical studies have reported that the levels of endogenous melatonin generally reduced in intestinal inflammation. Therefore, in view of the various protective effects of melatonin, more and more studies have focused on whether exogenous supplementation of melatonin could be used as an adjuvant treatment of intestinal inflammation, while it is not clear how to distinguish endogenous or exogenous supplements. The helpful effect of adjuvant melatonin therapy in IBD patients has been documented [32]. An increasing amount of evidence was collected from rodents to demonstrate that melatonin administration reduced colitis symptoms [27,28,29,33,34]. These effects were attributed to various mechanisms, including decreasing the level of inflammatory cytokines, inhibiting the activity of nuclear factor-kappa beta (NF-κB), reducing the production of matrix metalloproteinases or nitric oxide, regulating apoptosis and oxidative stress, modulating the attenuation of immunological injury by the activity of macrophages, and suppressing bacterial translocation [35,36,37,38]. However, no exact mechanism has illustrated the relationship among melatonin, obesity, and colitis. It is meaningful to address the role of melatonin in obesity-promoted colitis.

This study aims to examine the protective role of melatonin on HFD-promoted DSS-induced colitis in a mouse model, and we may expect to provide the potential for developing a novel therapy to prevent the progression of colitis in obese individuals.

## 2. Results

### 2.1. Obesity Aggravates Colitis Severity upon Dextran Sodium Sulfate (DSS) Challenge

**Figure 1:**
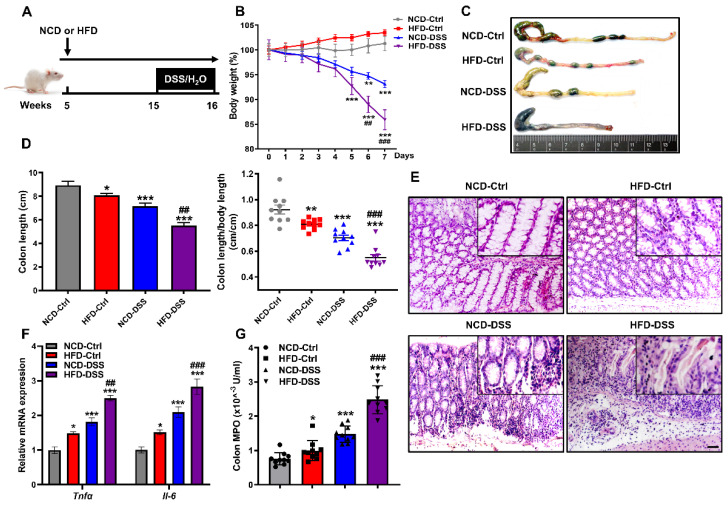
Obesity aggravates the severity of DSS-induced colitis. (**A**) Schematic diagram of DSS-induced experimental colitis in mice. Male CD1 mice were fed with NCD or HFD for 10 weeks. To induce colitis, mice were additionally given 3% DSS by drinking water for 7 days. Mice were sacrificed on day 7 with DSS treatment, and colons were collected for analysis. (**B**) Bodyweight changes. (**C**) Representative images of the colon’s appearance. (**D**) Statistical data of the colon length. (**E**) Hematoxylin and eosin (H&E) staining of colon sections. Scale bar = 50 μm. (**F**) The mRNA expression levels of the proinflammatory genes *Tnfα* and *Il-6* were measured by real-time PCR in the colon. (**G**) Colon MPO levels. Values are presented as the mean ± SEM. *n* = 10. * *p* < 0.05, ** *p* < 0.01, *** *p* < 0.001 compared with NCD-Ctrl; ## *p* < 0.01, ### *p* < 0.001 versus NCD-DSS.

Clinical evidence has shown that obesity and a high-fat diet (HFD) are associated with the severity of colitis [39,40]. To further confirm the effect of obesity on the development of colitis, male CD1 mice fed a chow diet (NCD) or HFD for 10 weeks were additionally subjected to DSS-induced colitis (Figure 1A). Changes in mouse body weight were recorded within 7 days after DSS induction. As expected, body weight loss with time upon the induction of DSS, while the percentage of weight loss in the HFD group was significantly higher than that in the NCD group (Figure 1B and Supplemental Figure S1). Consistent with the much higher body weight decrease percentage, colons were significantly shortened and swollen in the DSS mice fed the HFD (Figure 1C,D). Moreover, DSS installation resulted in histological damage, which was further aggravated by the HFD, as shown by mucosa damage and less villus structure (Figure 1E). Similarly, the expression of proinflammatory cytokines (*Tnfα* and *Il-6*) in the colon of obese mice treated with DSS was significantly upregulated (Figure 1F), and the activity of colonic myeloperoxidase (MPO), a marker of neutrophil activity, was significantly higher than that in NCD-DSS mice (Figure 1G). Therefore, these observations indicate that HFD exacerbates the severity of DSS-induced colitis.

### 2.2. Melatonin Alleviates DSS-Induced Colitis in Obese Mice

**Figure 2:**
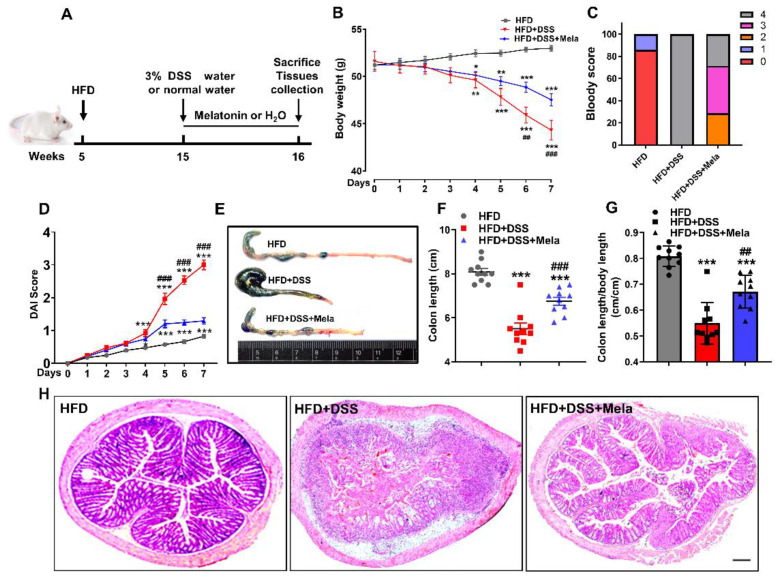
Melatonin alleviates DSS-induced colitis in obese mice. (**A**) Conceptual graph of the experimental procedure analyzing the effects of melatonin on DSS-induced colitis in obese mice. Male CD1 obese mice were treated daily with water or 10 mg/kg/day melatonin by intragastric gavage for 7 days. Tissues were collected on day 7 for analysis. (**B**) Bodyweight. (**C**)The gross bleeding was evaluated on day 7. (**D**) The DAI score was assessed every day after DSS water supplementation. (**E**) Representative images of the colon’s appearance. (**F**,**G**) Statistical data of the colon length. (**H**) Representative images of histological damage in the colon with the indicated treatment. Scale bar = 200 μm. Values are presented as the mean ± SEM. *n* = 10. * *p* < 0.05, ** *p* < 0.01, *** *p* < 0.001 compared with HFD; ## *p* < 0.01, ### *p* < 0.001 versus HFD + DSS.

Melatonin has been studied for potential benefits as a coadjuvant treatment in gastrointestinal diseases, especially irritable bowel syndrome (IBS), Crohn’s disease (CD), ulcerative colitis (UC), and necrotizing enterocolitis [28]. In the present study, we focused on determining whether melatonin has chemopreventive effects on HFD-propelled colitis in mice (Figure 2A). According to body weight curves, the HFD group remained stable during the experimental process, while the HFD + DSS group underwent substantial weight loss. However, melatonin administration alleviated this effect (Figure 2B), suggesting that DSS-induced malnutrition or exhaustion conditions were reversed by melatonin treatment. The disease activity index (DAI) is a comprehensive index that reflects the overall severity of colitis and is evaluated by body weight loss, stool consistency, and gross bleeding. It was observed that the HFD + DSS mice had a rather high DAI score and hematochezia, while treatment with melatonin helped reduce the DAI score, indicating that melatonin showed a better preventive ability to some extent (Figure 2C,D). Similarly, the length of the colon of obese mice treated with DSS was significantly shortened (Figure 2E–G), accompanied by the destruction of the integrity of the colon and the thickening of the gland structure (Figure 2H). In contrast, melatonin administration had the opposite effect. Interestingly, the ratio of spleen weight to body weight in the melatonin-treated mice significantly decreased compared to HFD + DSS mice, implying that melatonin treatment reduced DSS-induced spleen enlargement (Supplemental Figure S2). Collectively, these results suggested that melatonin could improve DSS-induced colitis in obese mice.

### 2.3. Melatonin Attenuates DSS-Induced Inflammation and Apoptosis in Obese Mice

**Figure 3:**
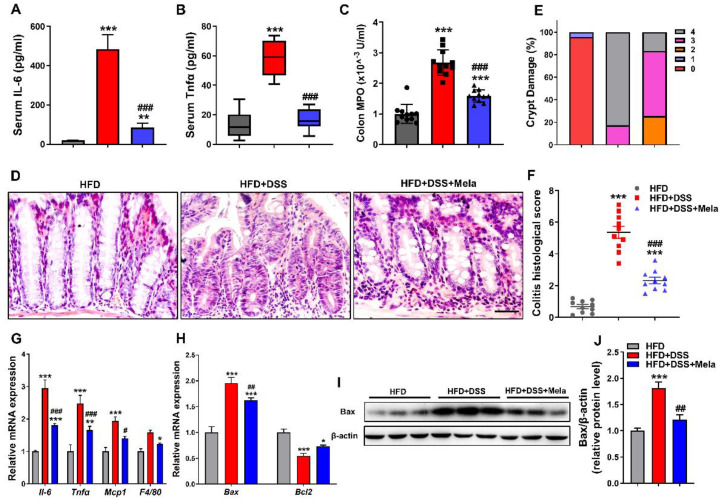
Melatonin attenuates DSS-induced inflammation and apoptosis in obese mice. (A and B) ELISAs of the production of serum IL-6 (**A**) and Tnfα (**B**). (**C**) MPO activity in mucosal lysates of mice. (**D**–**F**) Representative H&E staining (**D**), colitis histological score (**E**), and crypt damage (**F**) in colons. Scale bar  =  50 μm. (**G**,**H**) Real-time RT-PCR quantitation of colon proinflammatory cytokines (**G**) and apoptosis-associated genes (**H**). (**I**,**J**) Immunoblot analysis of Bax proteins in colon tissues (**I**) and quantification (**J**) in the three groups of mice. The data are expressed as the mean ± SEM. *n* = 10. * *p* < 0.05, ** *p* < 0.01, *** *p* < 0.001 compared with HFD; # *p* < 0.05, ## *p* < 0.01, ### *p* < 0.001 versus HFD + DSS.

Since we found that melatonin alleviates the systematic features of DSS-induced colitis in obese mice, we further investigated the anti-inflammatory and antiapoptotic activity of melatonin on DSS-induced bowel inflammation in obese mice. Although serum *Il-6* and *Tnfα* were significantly increased in the HFD + DSS group, treatment with melatonin markedly inhibited the production of these proinflammatory cytokines (Figure 3A,B). Melatonin supplementation also downregulated the activity of MPO compared with the HFD + DSS group (Figure 3C). Next, we focused on colon inflammation histologically. Hematoxylin and eosin (H&E) staining revealed that the colon mucosa of the HFD + DSS group had more severe inflammatory cell infiltration and glandular destruction than the HFD controls. However, melatonin treatment reduced the severity of the above manifestations, and histopathological scoring showed less inflammation and crypt damage in the colon (Figure 3D–F and Supplemental Figure S3), indicating that melatonin attenuated DSS-induced inflammatory injury. Mucosal injury in colitis causes cytokine and chemokine release from intestinal epithelial cells (IECs) in response to the development of colitis [41,42,43]. We observed that the mRNA levels of related inflammatory cytokines and chemokines (*Il-6*, *Tnfα*, *Mcp1*, and *F4/80*) in colonic tissues (Figure 3G) were markedly increased in the HFD + DSS group. At the same time, melatonin partially reduced the expression of inflammatory factors, suggesting that melatonin inhibited more severe colitis and excessive activation of the inflammatory response. Increased apoptosis has been revealed to be a pathogenic factor in the development of colitis [44,45]. Interestingly, melatonin treatment lightened the characteristics of cell apoptosis after DSS treatment, as evidenced by decreased *Bax* mRNA (Figure 3H) and protein expression (Figure 3I,J) and increased *Bcl2* mRNA (Figure 3H) expression compared with the HFD + DSS group, which indicates that the protection of cell apoptosis may be an important condition for the anti-inflammatory approach. Collectively, these findings indicate that melatonin exerts its protective effect on colitis development at least partially by suppressing the inflammatory and apoptosis response.

### 2.4. Melatonin Enhances Antioxidant Activities to Improve Obesity-Related Colitis

**Figure 4:**
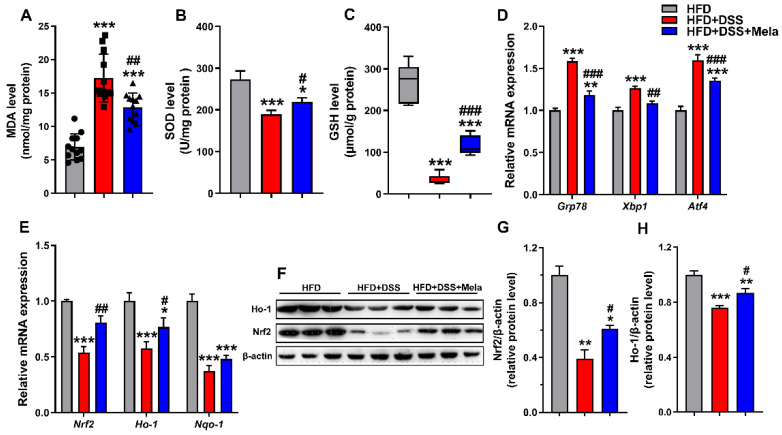
Melatonin enhances antioxidant activities to improve obesity-related colitis. (**A**–**C**) Colonic levels of MDA (**A**), SOD (**B**), and GSH (**C**) were examined by chemical chromatometry. (**D**,**E**) Colonic mRNA levels of ER stress markers (*Grp78*, *Xbp1* and *Atf4*) (**D**) and oxidative stress markers (*Nrf2*, *Nqo1* and *Ho-1*) (**E**) by qRT-PCR in the colon. (**F**–**H**) The protein expression of Ho-1 and Nrf2 in colonic tissues was assessed by Western blotting (**F**), and the relative protein intensity of Ho-1 (**G**) and Nrf2 (**H**) was normalized to that of β-actin on day 7. Data are expressed as the mean ± SEM. *n* = 10. * *p* < 0.05, ** *p* < 0.01, *** *p* < 0.001 compared with HFD; # *p* < 0.05, ## *p* < 0.01, ### *p* < 0.001 versus HFD + DSS.

Endoplasmic reticulum (ER) stress has been proven to be a pathological component of many chronic diseases, including colitis [46]. Previous studies have shown the mechanism by which obesity aggravates DSS-induced colitis by focusing on oxidative stress, which is considered an important driver of acute inflammation and apoptosis [47,48,49]. We further investigated the effect of melatonin on oxidative activity in DSS-treated obese mice. As expected, the HFD + DSS mice were characterized by a significant increase in colonic MDA (oxidative damage indicator) levels along with a concomitant decrease in SOD and GSH (antioxidant indicators) contents (Figure 4A–C). The increase in colonic MDA levels of the HFD + DSS group was prevented by melatonin (Figure 4A). Meanwhile, the tissue antioxidant enzymes (SOD and GSH) were higher after melatonin treatment (Figure 4B,C). Concomitantly with an increase in acute progressive intestinal inflammation, the HFD + DSS mice showed increased expression of ER stress marker genes (*Grp78*, *Xbp1*, and *Atf4*) (Figure 4D) and decreased levels of associated antioxidant enzymes (*Nrf2*, *Nqo-1*, and *Ho-1*) (Figure 4E–H) compared with the controls, indicating that elevated oxidative stress is related to DSS-mediated colitis in obese mice. However, intervention with melatonin caused a reduction in colitis-associated oxidative stress, as observed by a significant decrease in *Grp78*, *Xbp1*, and *Atf4* and an increase in *Nrf2*, *Nqo-1*, and *Ho-1* levels in the colon of mice (Figure 4D–H), substantiating the antioxidant effect of melatonin on DSS-mediated colitis in obese mice.

### 2.5. Melatonin Reverses Intestinal Barrier Integrity in DSS-Treated Obese Mice

**Figure 5:**
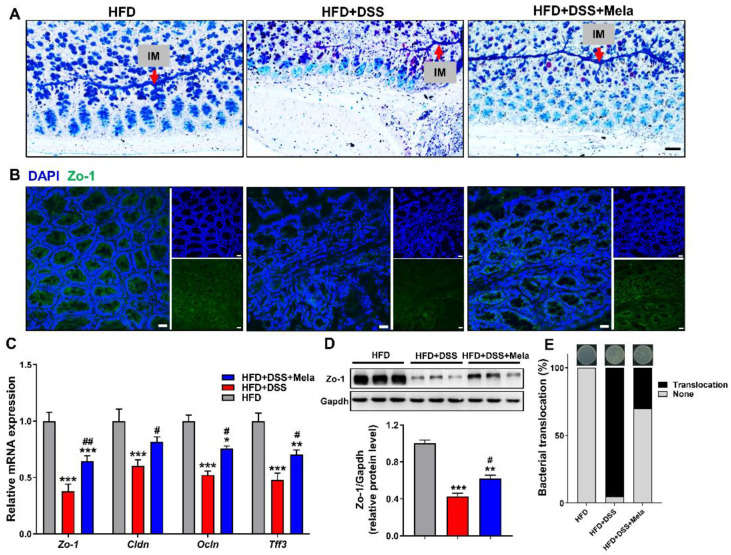
Melatonin reverses intestinal barrier integrity in DSS-treated obese mice. (**A**) Representative AB-PAS-stained colonic sections. Acid mucins: blue. Mixtures of acidic and neutral mucins: purple. Nuclei: pale blue. IM, inner mucus layer. (Scale bar = 50 μm). (**B**) Immunofluorescence of Zo-1 (**green**) and DAPI (**blue**) in colon sections (Scale bar = 50 μm). (**C**) The mRNA expression of *Zo-1*, *Cldn*, *Ocln*, and *Tff3* in the colon. (**D**) Zo-1 protein levels were measured by Western blotting, (**E**) Incidence of bacterial translocation. The results are mean ± SEM. *n* = 10. * *p* < 0.05, ** *p* < 0.01, *** *p* < 0.001 compared with HFD; # *p* < 0.05, ## *p* < 0.01 versus HFD + DSS.

The intestinal epithelial barrier plays a critical role in the development of colitis. Colonic goblet cells can produce and secrete mucus, which is essential for maintaining the colonic mucosal barrier and preventing luminal microbial invasion. Meanwhile, they could produce a large number of complex secreted proteins, which easily lead to a higher rate of protein misfolding in the ER. Given the induction of ER stress markers, we further determined whether HFD-induced inflammation and stress changed the mucosal barrier through histology and gene expression analysis. As shown in Figure 5A, we found that the obesity-related colitis model was accompanied by a decrease in the number of goblet cells and thinning of the mucous layer using AB-PAS staining in the colonic sections. However, melatonin treatment significantly improved the reduction in goblet cells and restored mucus (Figure 5A). In addition, as shown by immunofluorescence, the increased expression of tight junction protein Zo-1 further supported the improvement of the structural damage of the intestinal barrier in melatonin treatment (Figure 5B). We also detected the expression of markers of the tight junction structure. Interestingly, we observed that the mRNA levels of *Zo-1*, *Cldn*, *Ocln*, and *Tff3* were increased in the melatonin group. However, these levels were reduced in the HFD + DSS group (Figure 5C). Furthermore, Western blotting was conducted to evaluate the protein expression of Zo-1 in the colon. As shown in our results, the protein level of Zo-1 was upregulated by melatonin compared with the HFD + DSS group (Figure 5D). When the intestinal mucosa integrity is damaged, some bacteria migrate to nearby tissues, such as the liver and spleen, affecting the function of these tissues [50]. In our experiments, we found that the integrity of the colonic mucosa was severely damaged by DSS treatment, and the colonies migrated to the nearby liver. As expected, melatonin supplementation decreased bacterial translocation (Figure 5E). Combined, these results show that melatonin could repair the structural integrity damage of colon mucosa in HFD-promoted DSS-induced colitis, suggesting that it is beneficial in maintaining epithelial barrier function.

### 2.6. Melatonin Inhibits Lipolysis and Fatty Acid Transport in the White Adipose Tissues of DSS-Treated Obese Mice

**Figure 6:**
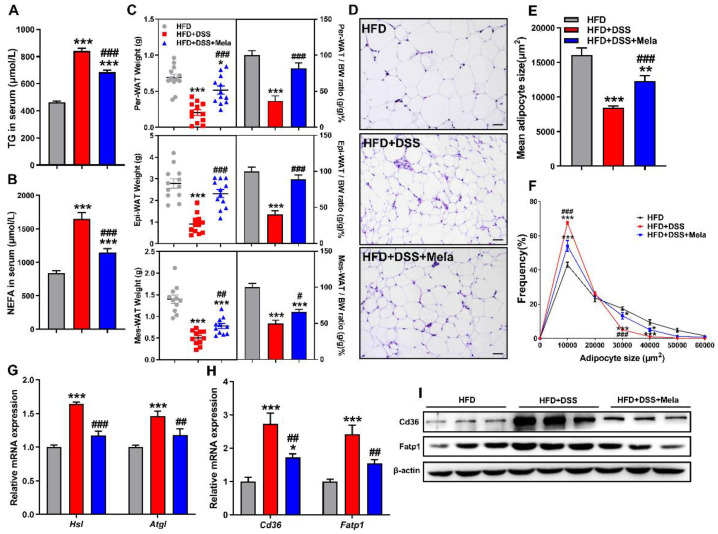
Melatonin inhibits lipolysis and fatty acid transport in the white adipose tissues of DSS-treated obese mice. (**A**,**B**) Serum concentrations of TC (**A**) and NEFA (**B**). (**C**) Per-WAT, Epi-WAT, and Mes-WAT weights and their respective ratios. (**D**) Representative H&E staining of Mes-WAT sections (scale bar = 100 μm). (**E**,**F**) Mean mesenteric adipocyte size (**E**) and adipocyte size frequency (**F**). (**G**,**H**) Mes-WAT mRNA expression of genes involved in lipolysis (*Hsl* and *Atgl*) (**G**) and lipid transport (*Cd36* and *Fatp1*) (**H**) was measured by real-time PCR analysis. (**I**) The Cd36 and Fatp1 protein levels were examined in Mes-WAT. Values are shown as the means ± SEM. *n* = 10. * *p* < 0.05, ** *p* < 0.01, *** *p* < 0.001 compared with HFD; # *p* < 0.05, ## *p* < 0.01, ### *p* < 0.001 versus HFD + DSS.

Colitis is related to nutritional deficiencies, and changes in energy metabolism might lead to increased lipid utilization [51,52]. Adipose tissue lipolysis is key to maintaining energy homeostasis by regulating triglycerides (TGs) and releasing free fatty acids (FFAs) into the circulation. Adipose tissue depots could be changed with intestinal inflammation [51,52,53]. Consistently, we also found robust inflammation in Mes-WAT in HFD + DSS mice (Supplementary Figure S4). Given the beneficial protective effects of melatonin, we hypothesized that melatonin plays a key role in adipose tissue lipolysis in a mouse model of colitis to participate in the local energy supply. Unsurprisingly, the serum levels of TG (Figure 6A) and nonesterified fatty acids (NEFAs) (Figure 6B) were higher in the HFD + DSS group and decreased in the melatonin group. Next, we paid attention to the weight of white adipose tissue, including perirenal white adipose tissue (Per-WAT), epididymal white adipose tissue (Epi-WAT), and mesenteric white adipose tissue (Mes-WAT). As shown in Figure 6C, the amounts and tissue ratio (tissue weights/body weight) of all three tissue types were significantly decreased by DSS treatment and were rescued by melatonin in obese mice. In addition, we analyzed the size of adipocytes obtained from Mes-WAT (Figure 6D). The HFD + DSS and melatonin-treated groups displayed decreased mean adipocyte size compared with the HFD group. However, the melatonin-treated mice revealed a mildly increased adipocyte size compared with HFD + DSS mice (Figure 6E). Combined with the analysis of the size distribution of adipocytes (Figure 6F), we could see that melatonin treatment specifically inhibited the lipolysis of adipocytes, suggesting that melatonin could inhibit increased lipid utilization in HFD-promoted DSS-induced colitis in mice. Based on this hypothesis, we examined the expression of lipolysis-related genes. Interestingly, the key genes (*Hsl* and *Atgl*) for lipolysis were significantly increased in the HFD + DSS group (Figure 6G), while the expression level in the melatonin-treated mice was relatively decreased. Furthermore, we focused on the expression of lipid transport markers. *Cd36* and *Fatp1* in the HFD + DSS mice were significantly upregulated, and melatonin treatment restored these lipid transport levels (Figure 6H,I). Taken together, these results suggest that colitis could promote lipolysis to provide sufficient energy for self-metabolism, while melatonin could slow down the process.

### 2.7. Melatonin Mediates Adipocyte-Induced Autophagy in DSS-Treated Obese Mice

**Figure 7:**
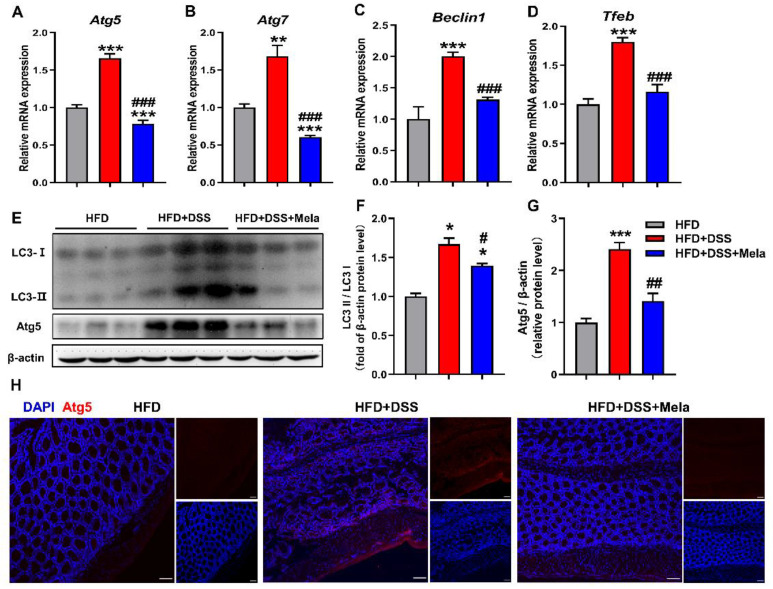
Melatonin mediates adipocyte-induced autophagy in DSS-treated obese mice. (**A**–**D**) Colonic mRNA expression of genes involved in autophagic genes (*Atg5*, *Atg7*, *Beclin1*, and *Tfeb*) was measured by real-time PCR analysis. (**E**–**G**) The LC3II/I and Atg5 protein levels were examined in the colon. (**H**) Immunofluorescence of Atg5 (**red**) and DAPI (**blue**) in colon sections (scale bar = 50 μm). Data in the graphs represent mean ± SEM. *n* = 10. Significant differences are shown by * *p* < 0.05, ** *p* < 0.01, *** *p* < 0.001 compared with HFD; # *p* < 0.05, ## *p* < 0.01, ### *p* < 0.001 versus HFD + DSS.

Autophagy is an evolutionarily conserved catabolic mechanism responsible for degrading various components in cells, including lipids, thereby promoting cell survival under energy stress conditions [54,55]. Given the finding that fatty acids released by adipocytes are easily transferred to adjacent inflammatory cells, we next determined whether the uptake of fatty acids stimulates autophagy in inflammatory intestinal epithelial cells to facilitate lipolysis. As shown in our data, the mRNA levels of autophagic genes (*Atg5*, *Atg7*, *Beclin1*, and *Tfeb*) were found to be remarkably increased in the HFD + DSS group compared with the HFD group (Figure 7A–D), suggesting that uptake of fatty acids triggers autophagy in obesity-related colitis. However, supplementation with melatonin reduced the stimulatory effect of DSS-induced autophagy in obese mice, as evidenced by decreased mRNA expression of the abovementioned autophagy marker genes (Figure 7A–D) and downregulated LC3II/I and Atg5 protein expression (Figure 7E–G). Furthermore, Atg5 expression was significantly blocked by melatonin in immunofluorescence staining assays (Figure 7H). The results from our study indicate that melatonin could modulate autophagy to slow down the adipocyte-mediated prosurvival advantage, as shown by diminished severity of intestinal damage.

## 3. Discussion

In this study, we investigated the role of melatonin in HFD-promoted DSS-induced experimental colitis in mice. We demonstrated that obesity promotes the process of colitis, and the same trend has been shown in other studies [56,57,58]. In our animal model, the protective effects of melatonin on HFD-promoted DSS-induced colitis were explained in terms of anti-inflammatory, antiapoptotic, antioxidant, and intestinal mucosal integrity. It has been reported that colitis is associated with nutritional deficiencies, and changes in energy metabolism may lead to increased lipid utilization [51,52]. Adipose tissue promotes FFA release or transport through lipolysis to maintain energy homeostasis [59]. In our research, we also found that obesity-induced colitis could promote lipolysis and fatty acid transport for self-energy metabolism. As an evolutionarily conserved catabolic mechanism, autophagy is responsible for the degradation of intracellular lipids, thereby promoting cell survival under energy stress conditions. The uptake of fatty acids may trigger autophagy in intestinal epithelial cells to support the survival of inflammatory or cancer cells [54,55]. Interestingly, we found that melatonin inhibited autophagy to reduce adipocyte-mediated survival advantages, which suggests that melatonin-targeted autophagy may provide benefits in the treatment of obese-promoted colitis.

Many studies have discussed the mechanism by which HFD-induced obesity aggravates colitis in mice. The mouse strains used in many experiments are different, such as C3H/HeJ, C3H/HeJBir, C57BL/6J and DBA/2J. It has been reported that different strains and substrains of mice have different susceptibility and responsiveness to DSS-induced colitis [60]. We decided to choose CD1 mice, because many other studies also use CD1 mice as animal models to study colitis [61,62,63,64,65,66]. Here, we established a mouse colitis model with 3% DSS to confirm the susceptibility of HFD to colitis. Mouse weight data showed that there was a significant difference between HFD and normal diet mice. However, after DSS-induced colitis, the bodyweight of HFD-fed mice became significantly reduced, the length of the colon was significantly shortened, and the severity of colitis was the highest. When an acute attack occurs, a large number of lymphocytes, macrophages, and activated white blood cells infiltrate into the inner lining of the intestinal mucosa [67,68]. Interestingly, we also found a small amount of disintegration of goblet cells, which maintained the structural integrity of the intestinal epithelium, and a small infiltration of inflammatory cells in NCD-fed DSS-induced colitis mice. However, in HFD-induced DSS-induced colitis mice, the glands were completely necrotic, and many inflammatory cells infiltrated. According to the observations during the mouse modeling period, the DSS water consumption and food intake of HFD-fed mice was less than that of NCD-fed mice, but the severity of colitis and histological score were higher than those of NCD-fed mice, indicating that the HFD has a significant impact on the occurrence of inflammation, which itself has a strong proinflammatory effect.

It is well known that increased levels of free radicals and decreased antioxidant capacity are characteristic of colitis [69,70]. Excessive inflammation under oxidative stress plays a key role in the pathogenesis of colitis [71,72,73,74]. HFD-induced inflammation and stress may increase the misfolding of secreted proteins in intestinal goblet cells, leading to alterations in intestinal epithelial barrier integrity. In addition, oxidative stress is considered the key driver of cell apoptosis [75]. Paracellular tight junction dysfunction and abnormal intestinal epithelial cell apoptosis may lead to epithelial barrier disruption, which is the pivotal pathological mechanism of colitis [76,77]. Our study showed that melatonin ameliorated more severe colonic injury and higher mucosal inflammatory factor expression in HFD-induced obese mice. An increase in progressive intestinal inflammation accompanied HFD, and the HFD + DSS group was characterized by a significant upregulation in colonic tissue MDA contents along with a concomitant downregulation in GSH and SOD levels. Meanwhile, the expression of genes (*Grp78*, *Xbp1*, and *Atf4*) that act as markers of ER stress was increased, and the levels of related antioxidant enzymes (*Nrf2*, *Nqo-1*, and *Ho-1*) were decreased in the HFD + DSS group. These results hypothesized that HFD-induced obesity exacerbated colitis in mice, which may be associated with increased oxidative stress. However, melatonin intervention resulted in a reduction in oxidative stress related to colitis, which in turn inhibited the activation of proapoptotic pathways and ultimately reduced the disruption of the colonic epithelial barrier, suggesting that melatonin intervention might be an important protective and treatment plan for obesity-related colitis.

Colitis has been reported to alter energy metabolism. Patients with colitis have a reduced intake of nutrients due to abdominal pain and anorexia. Mucosal inflammation and diarrhea lead to a loss of the absorption of nutrients, including proteins. Alterations in energy metabolism may accelerate increased energy expenditure and lipid utilization in patients with colitis [78,79]. Adipose tissue lipolysis is a key way to maintain energy balance through TG degradation and the release of FFAs into the blood circulation. As mentioned above, patients with colitis have fat mass depletion, which changes adipose tissue deposition [51,53]. In addition, the content of Epi-WAT and the size of adipocytes were decreased in DSS-induced colitis mice [52]. Previous studies have shown that melatonin is involved in the lipolysis of adipose tissue. However, melatonin-mediated lipolysis is controversial. Some studies have shown that melatonin promotes lipolysis [80,81,82,83,84], while other studies suggest the opposite [85,86]. The role of melatonin in adipose tissue lipolysis under inflammatory conditions has not been well elucidated. Our results indicated that melatonin may be regarded as a negative factor for lipolysis in DSS-induced colitis and would regulate the local energy supply.

Autophagy is a catalytic mechanism of evolutionary preservation that relates the degradation of cytoplasmic proteins, organelles, and lipids by lysosomes to facilitate the survival of cells under energy stress conditions [54,87]. Some studies have revealed that intracellular lipids stored as lipid droplets may be degraded and metabolized by autophagy and degradation of lysosomal mediators [88,89,90]. In addition, inflammation and oxidative stress were thought to be the underlying mechanisms that induce autophagy [91,92,93,94,95,96]. Autophagy induction has been reported by various pro-inflammatory cytokines, including NF-κB, Tnf-α, and interferon-γ [92,97]. It has already been reported that oxidative stress induces autophagy during nutritional deficiency, ischemia or reperfusion, hypoxia, and cellular stress [91,93,94,95]. Our findings were consistent with several other studies showing that autophagy-induced stimulation may promote inflammation, oxidative stress, and cellular lipolysis to meet energy requirements. Interestingly, melatonin intervention could modulate the process of autophagy to reduce inflammation and oxidative stress, and inhibiting autophagy reduced adipocyte-mediated prosurvival advantages, indicating that melatonin-mediated autophagy may provide protection for obesity-related colitis.

This study had some limitations. Data from recent years indicate that melatonin at a physiologically relevant concentration is closely related to the gut microbiota and inflammation [84,98,99,100]. The gut microbiota could be involved in melatonin synthesis and secretion [101], and melatonin also changes the microbiome of the GIT to regulate different physiological activities, such as preventing obesity [84,102,103]. It is widely believed that colitis involves a disturbance in the homeostasis between the gut microbiota and the host immune system [104], but the possible effects of melatonin on dysbiosis in colitis is not yet clear. Further study is also required to show how melatonin regulates the gut microbiota in colitis and to explain the interaction between melatonin, gut microbiota and obesity. Regulation of the gut hormone/gut microbiota axis would provide a promising target of interest for intestinal diseases in the future [105].

**Figure 8:**
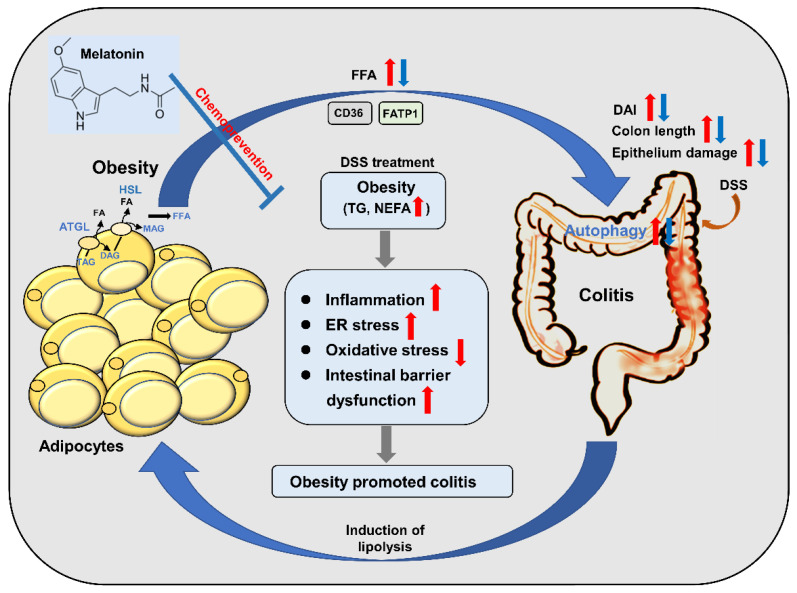
Schematic model depicting melatonin prevents colitis in obese individuals. HFD-promoted obesity accelerated DSS-induced colitis, while melatonin intervention improved colitis in mice. Melatonin significantly alleviated inflammation by increasing anti-inflammatory cytokine release and reducing the level of proinflammatory cytokines induced by HFD + DSS in mice. Furthermore, melatonin expressed antioxidant activities, resulting in improved colitis in HFD + DSS-treated mice. Moreover, melatonin reverses intestinal barrier integrity in DSS-treated obese mice. In addition, melatonin could reduce the ability of inflammatory cells to utilize fatty acids and decrease the growth-promoting effect of lipids by inhibiting autophagy. Taken together, the results indicate that the inhibitory effect of melatonin on autophagy weakens the lipid-mediated prosurvival advantage, which suggests that melatonin-targeted autophagy may provide a unique opportunity to prevent obesity-associated colitis. The red arrows represent the role of the HFD + DSS group. The blue arrow represents the action of the melatonin group.

Taken together, our results suggest that HFD-promoted DSS-induced colitis increases intestinal oxidative stress and inflammation, affecting autophagy and intestinal cell proliferation and reducing the expression of tight junction proteins, leading to loss of intestinal mucosal integrity. However, melatonin intervention alleviated DSS-induced inflammation, intestinal barrier dysfunction, and antioxidative stress. In addition, melatonin could reduce the ability of inflammatory cells to utilize fatty acids and decrease the growth-promoting effect of lipids by inhibiting autophagy. A diagrammatic representation depicting the melatonin-mediated protective mechanisms against obesity-associated colitis is shown in Figure 8. Our findings suggest that targeting autophagy may provide novel insight into obesity-induced colitis biology, offer new interventions for preventing colitis, and contribute to a better understanding of the mechanisms that regulate the various beneficial effects of melatonin. Our findings provide valuable insight for future research in this field. To fully understand the effects of melatonin on anti-colitis, further research with in vivo models is required to analyze the possible mechanisms of these properties. Taken together, our findings suggest that melatonin has the potential to be a novel therapeutic and chemopreventive agent, which could be used in the treatment of obesity-aggravated colitis.

## 4. Materials and Methods

### 4.1. Animals and Experimental Design

Five-week-old male CD1 (ICR) mice were purchased from Vital River Laboratory Animal Technology Co. Ltd. (Beijing, China). They were randomly housed five per cage in a controlled environment of temperature (25 ± 2 °C) and relative humidity (RH) (50 ± 60%) with a 12-h light-12-h dark cycle. The mice had ad libitum access to water and different diets for 10 weeks. Both the normal chow diet (NCD) and HFD (containing 60% kcal from fat, as shown in Supplementary Table S1) were purchased from Beijing HFK Bioscience Co. Ltd. (Beijing, China). Then, the body weight-matched mice were randomly distributed into five groups (*n* = 10–15). In the DSS-induced colitis model, the mice were treated with 3% DSS (*w*/*v*) in drinking water for 7 days. The HFD + DSS + Mela group was fed an HFD with melatonin at 10 mg/kg body weight by gavage once daily. All appropriate measures were taken to minimize animal suffering. All experimental animal protocols were performed in accordance with the guidelines approved by the Ethics and Animal Welfare Committee of Beijing Normal University (Approval No. CLS-EAW-2019-002).

### 4.2. Disease Activity Index (DAI)

DAI scores were conducted daily by unaware investigators following Cooper’s modified method to estimate the severity of experimental colitis [106]. These included body weight loss, stool consistency, and gross bleeding. Each score was quantified as follows: bodyweight loss: 0 (none), 1 (1–5%), 2 (5–10%), 3 (10–20%), and 4 (>20%); stool consistency: 0 (normal), 1 (loose stool), and 2 (diarrhea); stool blood: 0 (negative), 1 (weakly positive), 2 (positive), and 3 (strongly positive).

### 4.3. Fecal Occult Blood Testing

Fecal morphology was recorded, and the severity of fecal occult blood was detected by a urine fecal occult blood test kit (Nanjing Jiancheng Bioengineering Institute, Nanjing, China). A small amount of stool sample was smeared on the slides. Orthotolidine and hydrogen peroxide reagents were added to the surface of the stool samples. The results were as follows: negative (−): the samples do not develop color within 3 min; weak positive (+): the samples appear blue within 30 to 60 s; positive (++): the samples show bluish-green immediately; and strongly positive (+++): samples appear mazarine immediately.

### 4.4. Myeloperoxidase (MPO) Activity Assay

The activity of MPO in colon tissues of mice was detected by an MPO kit (Nanjing Jiancheng Bioengineering Institute, Nanjing, China). Briefly, colon tissue was homogenized in 50 mmol/L potassium phosphate buffered saline (PBS, pH 6.0) at 1:19, including 0.5% hexadecyltrimethylammonium hydroxide. The supernatant reacted with the mixed solution including 3,3′-dimethoxybenzidine and hydrogen peroxide (H_2_O_2_), pH 6.0. MPO activity was evaluated by analyzing the H_2_O_2_-dependent oxidation of 3,3′-dimethoxybenzidine.

### 4.5. Histological Analysis

To make paraffin sections, the colon and Mes-WAT were fixed in Carnoy’s solution for 2 h or 4% paraformaldehyde, paraffin-embedded, and sectioned at 5 µm following hematoxylin and eosin (H&E) and alcian blue periodic acid Schiff (AB-PAS) staining. H&E staining was performed according to standard methods as we previously used [107], briefly, hematoxylin solution for 3 min and eosin solution for 1 min. For the histological score of colitis, H&E staining colon sections were scored by an individual blinded to the details using a previously published system as indicated [106] (range 0–3), including crypt architecture, degree of inflammatory cell infiltration, muscle thickening, and goblet cell depletion. The histological score of colitis is the sum of each mouse. For AB-PAS staining, sections were stained with an AB-PAS stain kit (Beijing Solarbio Science and Technology Co., Ltd., Beijing, China).

### 4.6. Biochemical Analysis

According to the manufacturer’s instructions, the serum levels of IL-6 and Tnfα were measured using ELISA kits (NeoBioscience Technology Co., Ltd., Beijing, China). The OD values of absorbance at 450 nm were examined by a microplate reader. TG and NEFA in serum were determined using commercially available kits from Applygen Technologies Inc. (Beijing, China).

### 4.7. Antioxidant Activity Assay

SOD activity in colon tissue was assessed using a SOD assay kit (Nanjing Jiancheng Bioengineering Institute). It is based on the autooxidation of hydroxylamine. SOD activity is expressed as the value measured by the microplate reader at 450 nm.

GSH activity was determined using a commercially available kit (Nanjing Jiancheng Bioengineering Institute). According to the manufacturer’s instructions, GSH could react with dithionitrobenzoic acid (DTNB) to produce a yellow compound. The content of GSH could be quantitatively determined at 405 nm with a spectrophotometer.

### 4.8. MDA Activity Assay

The MDA level in colon tissue was determined by the velocity method using an MDA assay kit (Nanjing Jiancheng Bioengineering Institute, Nanjing, China). It is based on the reactivity of thiobarbituric acid (TBA). The procedures were carried out in accordance with the manufacturer’s protocols. Briefly, colon tissue was homogenized with trichloroacetic acid and centrifuged. The collected supernatant reacted with TBA. The resulting reaction was assessed by spectrophotometry at 532 nm.

### 4.9. Colony Forming Units Measurement

Bacterial translocation was assessed in tissues as previously described [50]. The liver was collected, weighed, and homogenized with PBS into suspension. Bacterial colony-forming units (CFU) from tissue samples were determined via serial dilutions on Luria broth agar. Colonies were counted after incubation at 37 °C for 48 h.

### 4.10. Gene Expression Analysis

Using an RNAprep Pure tissue kit (Tiangen, Beijing, China), total RNA was extracted from mouse tissues. A total of 2 μg of total RNA was reverse transcribed into cDNA with FastKing gDNA Dispelling RT SuperMix (Tiangen, Beijing, China). For RNA quantification, SYBR Green qPCR SuperMix (Transgen Biotech, Beijing, China) was performed using an ABI Q6 instrument (Thermo Fisher Scientific, Waltham, MA, USA) following the manufacturer’s instructions. The mRNA expression levels were normalized to *Gapdh* expression by the 2^−ΔΔCt^ value method. Information on primer sequences is listed in Supplementary Table S2.

### 4.11. Western Blot Analysis and Immunofluorescence

Tissue protein samples were harvested using RIPA lysis buffer (Applygen Technologies Inc., Beijing, China) supplemented with 1 mM PMSF (Sigma Aldrich, St. Louis, MO, USA). The concentration of proteins was assayed using a BCA protein assay kit (Thermo Fisher Scientific, Waltham, MA, USA). Western blot analysis was performed using a standard process as we previously used [108]. The following primary antibodies were used: Ocln, Zo-1, and β-actin. The band intensity was analyzed using ImageJ software. For immunofluorescence (IF), sections were incubated with antibodies against Zo-1 and Atg5 overnight in a humidity chamber at 4 °C, coimmunostained with anti-mouse 488- or 549-conjugated secondary antibody, and counterstained with DAPI. Information on the antibodies used is provided in Supplementary Table S3.

### 4.12. Statistical Analysis

All analyses were assessed using Prism 8.0 software (GraphPad Software Inc., San Diego, CA, USA). Experimental data are expressed as the mean  ± standard error (SEM). Differences between groups were analyzed using a two-tailed Student’s *t*-test. Differences among multiple groups were assessed by one-way analysis of variance (ANOVA). A value of *p*  ≤  0.05 was considered statistically significant.

## Data Availability

Data is contained within the article and supplementary material.

## Supplementary Materials

The following are available online at https://www.mdpi.com/article/10.3390/ph14080822/s1: Figure S1: Body weight. Figure S2: Representative images of spleen and spleen weight. Figure S3: Representative H&E staining in colons. Figure S4: Effect of melatonin on the productions of cytokines of Mes-WAT in obese mice. Table S1: Compositions of experimental diets. Table S2: Sequences of real-time PCR primers. Table S3: List of antibodies used in the study.
