# Supplementary material for: Melatonin Attenuates Dextran Sodium Sulfate Induced Colitis in Obese Mice
Source: Pharmaceuticals (Basel). 2021 Aug 21;14(8):822. doi: 10.3390/ph14080822 (PMC8399719; doi:10.3390/ph14080822)
Supplement: Supplementary file 1 [file pharmaceuticals-14-00822-s001.zip › pharmaceuticals-1322262-supplementary.pdf]

Supplementary Figures

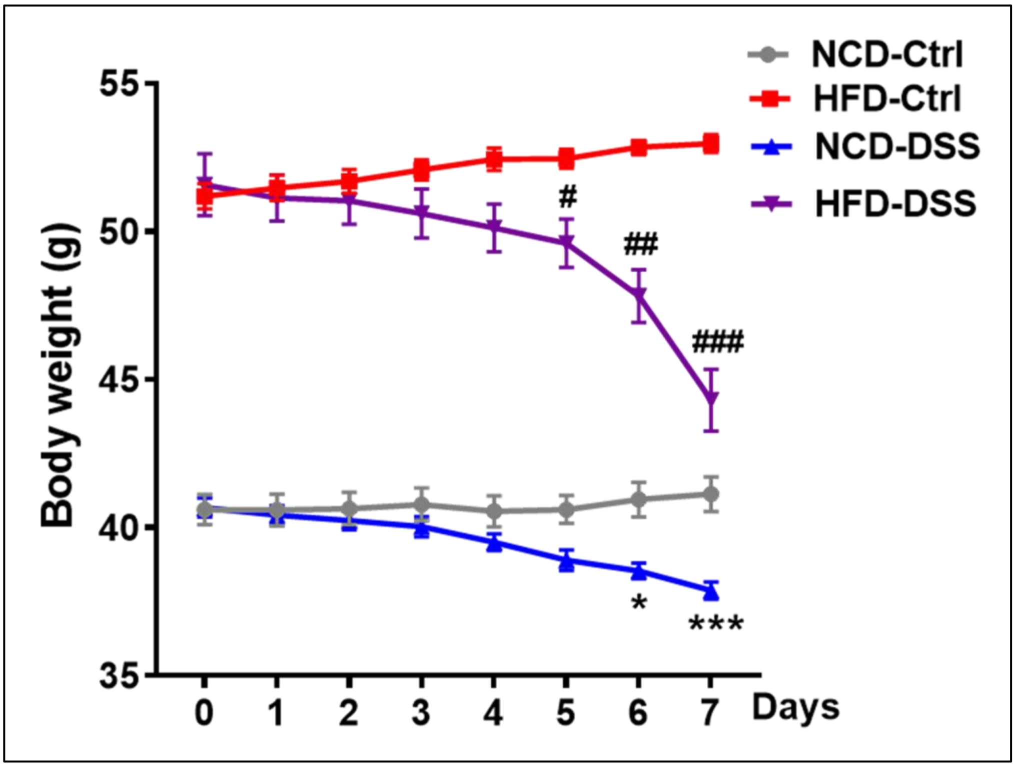

Figure S1. Body weight change in each group. Data are expressed as mean±SEM . * $p < 0.05$, *** $p < 0.001$ compared with NCD-Ctrl; # $p < 0.05$, ## $p < 0.01$, ### $p < 0.001$ versus HFD-Ctrl.

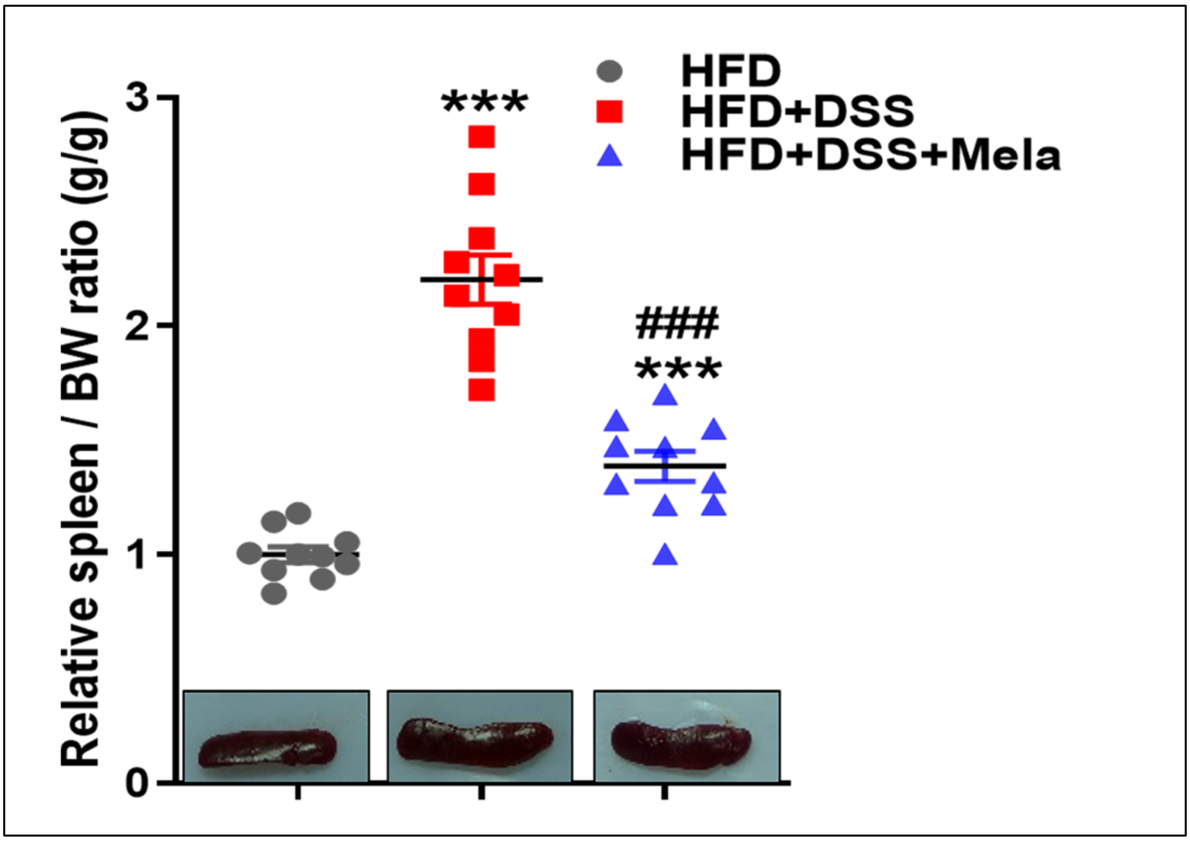

Figure S2. Representative images of spleen and spleen weight in each group. Data are expressed as mean±SEM . *** $p < 0.001$ compared with HFD; ### $p < 0.001$ versus HFD+DSS.

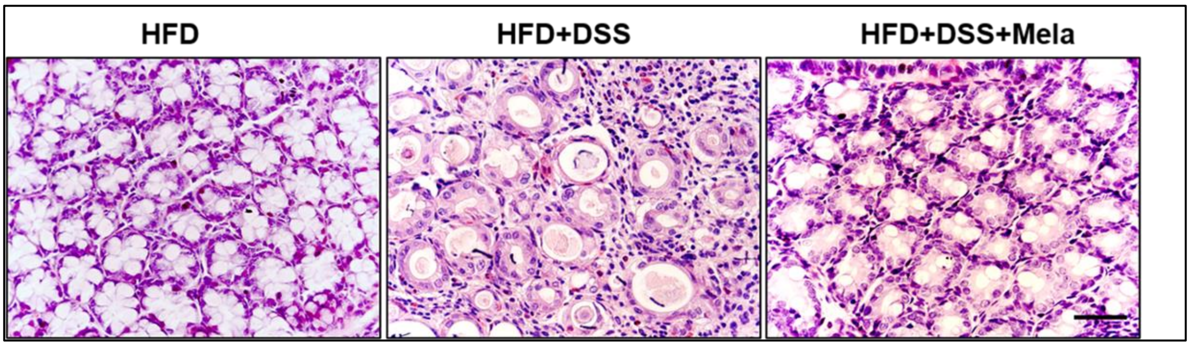

Figure S3. Representative H&E staining in colons (Scale bar = 50 μm) .

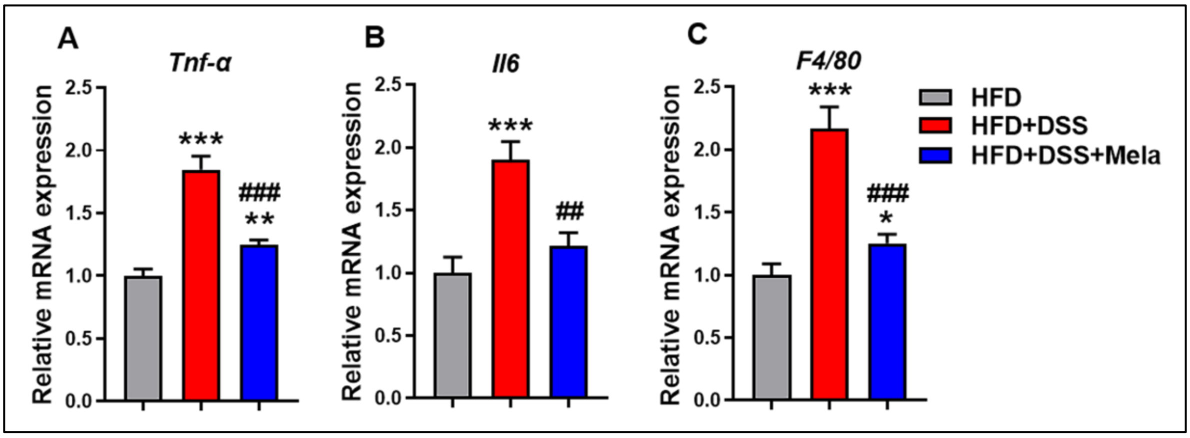

Figure S4. Effect of melatonin on the productions of cytokines of Mes-WAT in obese mice. (A-C) Expression of Tnfα(A), Il6 (B), F4/80 (C) in Mes-WAT. Data are expressed as mean±SEM of at least triplicated experiments from 3-5 mice. * $p < 0.05$, ** $p < 0.01$, *** $p < 0.001$ compared with HFD; ## $p < 0.01$, ### $p < 0.001$ versus HFD+DSS.

Supplementary Tables

Supplementary Table S1. Compositions of experimental diets.

	NCD		HFD (60% fat)	
	gm%	kcal%	gm%	kcal%
**Protein**	19.2	20	26.2	20
**Fat**	4.3	10	34.9	60
**Carbohydrate**	67.3	70	26.3	20

Supplementary Table S2. Sequences of real-time PCR primers.

Target gene	Sequence (5'-3')	Product Length
Il-6	F-CGACGGCCTTCCCTACTT	60
	R-TGGGAGTGGTATCCTCTGTGAA	
Tnfα	F-CATCTTCTCAAAATTCGAGTGACAA	175
	R-TGGGAGTAGACAAGGTACAACCC	
Mcp1	F-CAACTCTCACTGAAGCCAGCTCT	69
	R-CAGGCCCAGAAGCATGACA	
F4/80	F-GCTGTGAGATTGTGGAAGCA	166
	R-ATGGCCAAGGCAAGACATAC	
Bax	F-TTGCCCTCTTCTACTTTGCTAG	81
	R-CCATGATGGTTCTGATCAGCTC	
Bcl2	F-GATGACTTCTCTCGTCGCTAC	156
	R-GAACTCAAAGAAGGCCACAATC	
Grp78	F-ATGATGAAGTTCACTGTGGTGG	174
	R-CTGATCGTTGGCTATGATCTCC	
Xbp1	F-TTGCCTCTTCAGATTCTGAGTC	85
	R-GGGGAAGGACATTTGAAAAACA	
Atf4	F-AGTTTAGAGCTAGGCAGTGAAG	135
	R-CATACAGATGCCACTGTCATTG	
Nrf2	F-CAGCCATGACTGATTTAAGCAG	107
	R-CAGCTGCTTGTTTTCGGTATTA	
Ho-1	F-TCCTTGTACCATATCTACACGG	198
	R-GAGACGCTTTACATAGTGCTGT	
Nqo1	F-GAAGACATCATTCAACTACGCC	179
	R-GAGATGACTCGGAAGGATACTG	
Zo-1	F-CTGGTGAAGTCTCGGAAAAATG	97
	R-CATCTCTTGCTGCCAAACTATC	
Cldn	F-TTATGGTCATCAGCATCATCGT	111

	R-TGATCATGATCTTGGCCTTGAC	
Ocln	F-TGCTTCATCGCTTCCTTAGTAA<br>R-GGGTTCACTCCCATTATGTACA	155
Tff3	F-CTCTGGGATAGCTGCAGATTAC<br>R-TCAAAATGTGCATTCTGTCTCC	193
Hsl	F-CTCACAGTTACCATCTCACCTC<br>R-GATTTTGCCAGGCTGTTGAGTA	122
Atgl	F-CAGAGATGGACTTCGATTCCTT<br>R-CAGGTGCTCTAGAATTCGATCT	181
Cd36	F:GGAACTGTGGGCTCATTGC<br>R:CATGAGAATGCCTCCAAACAC	68
Fatp1	F:CGCTTTCTGCGTATCGTCTG<br>R:GATGCACGGGATCGTGTCT	120
Atg5	F-GATGGGATTGCAAAATGACAGA<br>R-GAAAGGTCTTTCAGTCGTTGTC	133
Atg7	F-TGTATAACACCAACACACTCGA<br>R-GGCAGGATAGCAAAACCAATAG	197
Beclin1	F-TAATAGCTTCACTCTGATCGGG<br>R-CAAACAGCGTTTGTAGTTCTGA	217
Tfeb	F-CACAGGTTACCCCGATACC<br>R-AGGGAGTCATCTAGGAGCATTA	96
Gapdh	F-GTCGTGGATCTGACGTGCC<br>R-TGCCTGCTTCACCACCTTCT	72

Supplementary Table S3. List of antibodies used in the study

Antigen name	Company	Identifier	Application	Dilution
β-actin	Sigma	A1978	WB	1:5000
Nrf2	proteintech	16396-1-AP	WB	1:1000
Ho-1	proteintech	10701-1-AP	WB	1:1000
Zo-1	proteintech	21773-1-AP	WB/IF	1:5000/1:1000
Atg5	proteintech	10181-2-AP	WB/IF	1:1000/1:100
LC3	proteintech	14600-1-AP	WB	1:1000
Bax	proteintech	50599-2-Ig	WB	1:5000
CD36	abcam	ab133625	WB	1:1000
Fatp1	Novus	H00376497-D01P	WB	1:1000

WB, Western blot;
IF,Immunofluorescence
